# Whole genomes from bacteria collected at diagnostic units around the world 2020

**DOI:** 10.1038/s41597-023-02502-7

**Published:** 2023-09-16

**Authors:** Sidsel Nag, Gunhild Larsen, Judit Szarvas, Laura Elmlund Kohl Birkedahl, Gábor Máté Gulyás, Wojchiech Jakub Ciok, Timmie Mikkel Lagermann, Silva Tafaj, Susan Bradbury, Peter Collignon, Denise Daley, Victorien Dougnon, Kafayath Fabiyi, Boubacar Coulibaly, René Dembélé, Georgette Nikiema, Natama Magloire, Isidore Juste Ouindgueta, Zenat Zebin Hossain, Anowara Begum, Deyan Donchev, Mathew Diggle, LeeAnn Turnbull, Simon Lévesque, Livia Berlinger, Kirstine Kobberoe Sogaard, Paula Diaz Guevara, Carolina Duarte Valderrama, Panagiota Maikanti, Jana Amlerova, Pavel Drevinek, Jan Tkadlec, Milica Dilas, Achim Kaasch, Henrik Torkil Westh, Mohamed Azzedine Bachtarzi, Wahiba Amhis, Carolina Elisabeth Satán Salazar, JoséEduardo Villacis, Mária Angeles Dominguez Lúzon, Dámaris Berbel Palau, Claire Duployez, Maxime Paluche, Solomon Asante-Sefa, Mie Moller, Margaret Ip, Ivana Mareković, Agnes Pál-Sonnevend, Clementiza Elvezia Cocuzza, Asta Dambrauskiene, Alexandre Macanze, Anelsio Cossa, Inácio Mandomando, Philip Nwajiobi-Princewill, Iruka N. Okeke, Aderemi O. Kehinde, Ini Adebiyi, Ifeoluwa Akintayo, Oluwafemi Popoola, Anthony Onipede, Anita Blomfeldt, Nora Elisabeth Nyquist, Kiri Bocker, James Ussher, Amjad Ali, Nimat Ullah, Habibullah Khan, Natalie Weiler Gustafson, Ikhlas Jarrar, Arif Al-Hamad, Viravarn Luvira, Wantana Paveenkittiporn, Irmak Baran, James C. L. Mwansa, Linda Sikakwa, Kaunda Yamba, Rene Sjogren Hendriksen, Frank Moller Aarestrup

**Affiliations:** 1 National Food Institute, Technical University of Denmark, Kemitorvet, Kgs, Lyngby, 2800 Denmark; 2 Microbiology Department, University Hospital “Shefqet Ndroqi”, Rruga Dr. Shefqet Ndroqi. Sauk, Tirana, 1044 Albania; 3 Microbiology Department, Canberra Hospital, Gilmore Cresent, Garran, 2605 Australian Capital Territory Australia; 4 Department of Microbiology, PathWest Laboratory Medicine, Fiona Stanley Hospital, 9 Robin Warren Drive, Murdoch, 6150 Western Australia Australia; 5 Research Unit in Applied Microbiology and Pharmacology of Natural Substances, Polytechnic School of Abomey-Calavi, University of Abomey-Calavi, 01 PO Box, Abomey-Calavi, 2009 Cotonou, Benin; 6 Department of Laboratory, Nouna Health Research Centre, Rue Namory Keita, Nouna, Burkina Faso; 7 Training and Research Unit in Applied Sciences and Technologies/Biochemistry-microbiology, University of Dedougou, Dedougou, 176 Boucle du Mouhoun Burkina Faso; 8 Clinical Research Unit of Nanoro, National Institutes of Medical Research, Ouagadougou, 176 Burkina Faso; 9 Department, University of Joseph KI-ZERBO, Street, Ouagadouogou, Burkina Faso; 10 Department of Microbiology, University of Dhaka, Dhaka, 1000 Bangladesh; 11 Clinical Laboratory of Microbiology and Virology, University Hospital “Lozenetz”, Str. Kozyak 1, Sofia, 1407 Sofia, Bulgaria; 12 Alberta Precision Laboratories, Alberta, Canada; 13 Service de microbiologie, Centre Integré Universitaire de Santé et de services sociaux de l’Estrie - Centre Hospitalier Universitaire de Sherbrooke, 3001 12è avenue Nord, Sherbrooke, J1H 5N4 Québec, Canada; 14 Department, Bioanalytica AG, Luzern, 6006 Switzerland; 15 Division of Clinical Bacteriology and Mycology, University Hospital Basel, Petersgraben 4, Basel, 4031 Switzerland; 16 Microbiology Group, Instituto Nacional de Salud, Avenida Calle 26·51-20 CAN, Bogotá, 111321 Colombia; 17 Charalampous, Microbiology Department, National Reference Laboratory for Antimicrobial Resistance Surveillance, Nicosia General Hospital, 215, Paleos Dromos Lefkosia-Lemesos str., Strovolos, 2029 Nicosia, Cyprus; 18 Department of Microbiology, University Hospital in Plzen, Edvarda Benese 1128/13, Plzen, 305 99 Czech Republic; 19 Department of Medical Microbiology, Motol University Hospital, V Uvalu 84, Prague, 15006 Czech Republic; 20 Otto-von-Guericke University, Magdebourg, Germany; 21 Klinisk Mikrobiologisk Afdeling, Hvidovre Hospital, Kettegårds Allé, Hvidovre, 2650 Denmark; 22 Laboratoire de Microbiologie Clinique, Centre Hospitalo-universitaire, 1 place du 1er Mai 1945, Algiers, 16000 Algeria; 23 National Reference Center for Antimicrobial Resistance, National Institute of Public Health Research “Dr. Leopoldo Izquieta Pérez”, Iquique N14-285, Quito, 170403 Pichicha Ecuador; 24 Centro de Investigación para la Salud en América Latina (CISeAL), Pontificia Universidad Católica del Ecuador, Quinto, 1701-2184 Pichincha Ecuador; 25 Department of Pathology and Experimental Therapy, Universitat de Barcelona, Barcelona, Spain; 26 Microbiology Department, Hospital de Bellvitge, Barcelona, 10587 Spain; 27 Institute of Microbiology, Centre Hospitalier Universitaire de Lille, Rue du Pr. Jules Leclercq, Lille, 59037 France; 28 Bacteriology laboratory, Centre hospitalier de Valenciennes, Avenue Désandrouin, Valenciennes, 59300 France; 29 Sekondi Public Health Laboratory, Ghana Health Service, Effia Nkwanta Regional Hospital, Effia Nkwanta Regional Hospital, Takoradi, Ghana; 30 Dronning Ingrids Hospital, Nuuk, Greenland; 31 Chinese University of Hong Kong, Shatin, Hong Kong; 32 Department of Clinical and Molecular Microbiology, University Hospital Centre Zagreb, Kišpatićeva 12, Zagreb, 10000 Croatia; 33 Medical Microbiology and Immunology, University of Pecs Medical School, Szigeti ut 12, Pecs, 7631 Hungary; 34 Department of Medicine and Surgery, University of Milano-Bicocca, Milan, Italy; 35 Laboratory Medicine Department, Hospital of Lithuanian University of Health Sciences Kauno klinikos, Eiveniu Str. 2, Kaunas, 50161 Lithuania; 36 Centro de Investigação em Saúde de Manhiça, Manhiça, Mozambique; 37 National Hospital Abuja, Abuja, Nigeria; 38 Faculty of Pharmacy, University of Ibadan, Ibadan, Oyo State Nigeria; 39 College of Medicine, University of Ibadan, Ibadan, Oyo State Nigeria; 40 University College of Ibadan, Ibadan, Oyo State Nigeria; 41 Obafemi Awolowo University, Ile-Ife, Nigeria; 42 Department of Microbiology and Infection Control, Akershus University Hospital, Sykehusveien 25, Lørenskog, 1478 Norway; 43 Southern Community Laboratories, University of Otago, 472 George Street, Otago, 9016 Dunedin, New Zealand; 44 Department of Industrial Biotechnology, Atta-ur-Rahman School of Applied Biosciences, National University of Sciences and Technology (NUST), H-12, Islamabad, 44000 Pakistan; 45 Molecular Diagnostic Section, Khyber Teaching Hospital (KTH), University Road, Peshawar, 25120 Pakistan; 46 Departamento de Bacteriologia, Laboratorio Central de Salud Publico, Avenida Venezuela y Tte Escurra, Asunción, CP 1429 Paraguay; 47 Basic Medical Sciences Department, Arab American University, AAUP st., Zababdeh, P240 Jenin, Palestine; 48 Division of Clinical Microbiology, Qatif Central Hospital, 3213 Dharan-Jubail Expressway, Al-Qatif, 32654-7376 Eastern Province Saudi Arabia; 49 Department of Clinical Tropical Medicine, Faculty of Tropical Medicine, Mahidol University, Ratchawithi Road, Bangkok, 10400 Thailand; 50 Department of Medical Sciences, National Institute of Health, Sariburi, Thailand; 51 Medical Microbiology Department, Karadeniz Technical University Farabi Hospital, Farabi Hastanesi, Trabzon, 61080 Ortahisar Turkey; 52 Lusaka Apex Medical School, Lusaka, Zambia; 53 Levy Mwanawasa Teaching Hospital, Lusaka, Zambia; 54 University Teaching Hospital, Lusaka, Zambia

**Keywords:** Infection, Antimicrobial resistance, Mobile elements, Developing world, Genetic databases

## Abstract

The Two Weeks in the World research project has resulted in a dataset of 3087 clinically relevant bacterial genomes with pertaining metadata, collected from 59 diagnostic units in 35 countries around the world during 2020. A relational database is available with metadata and summary data from selected bioinformatic analysis, such as species prediction and identification of acquired resistance genes.

## Background & Summary

Acquiring resistance-conferring genes is one of a number of mechanisms that can cause bacterial pathogens to become resistant to antimicrobial therapies^1^. Resistance genes can be located either chromosomally or on mobile genetic elements, such as plasmids^2^. Mobile genetic elements, in turn, can be horizontally transferred within bacterial communities and therefore play a key role in the geographic spread of antimicrobial resistance (AMR). Surveillance and monitoring of antimicrobial resistance are of high priority in many national and supra-national health organisations^3–8^. These efforts are highly motivated by a need to assess the size of the AMR problem, and help provide policy guidance on how to best ensure effective treatment and limit the further spread and development of AMR.

**Fig. 1:**
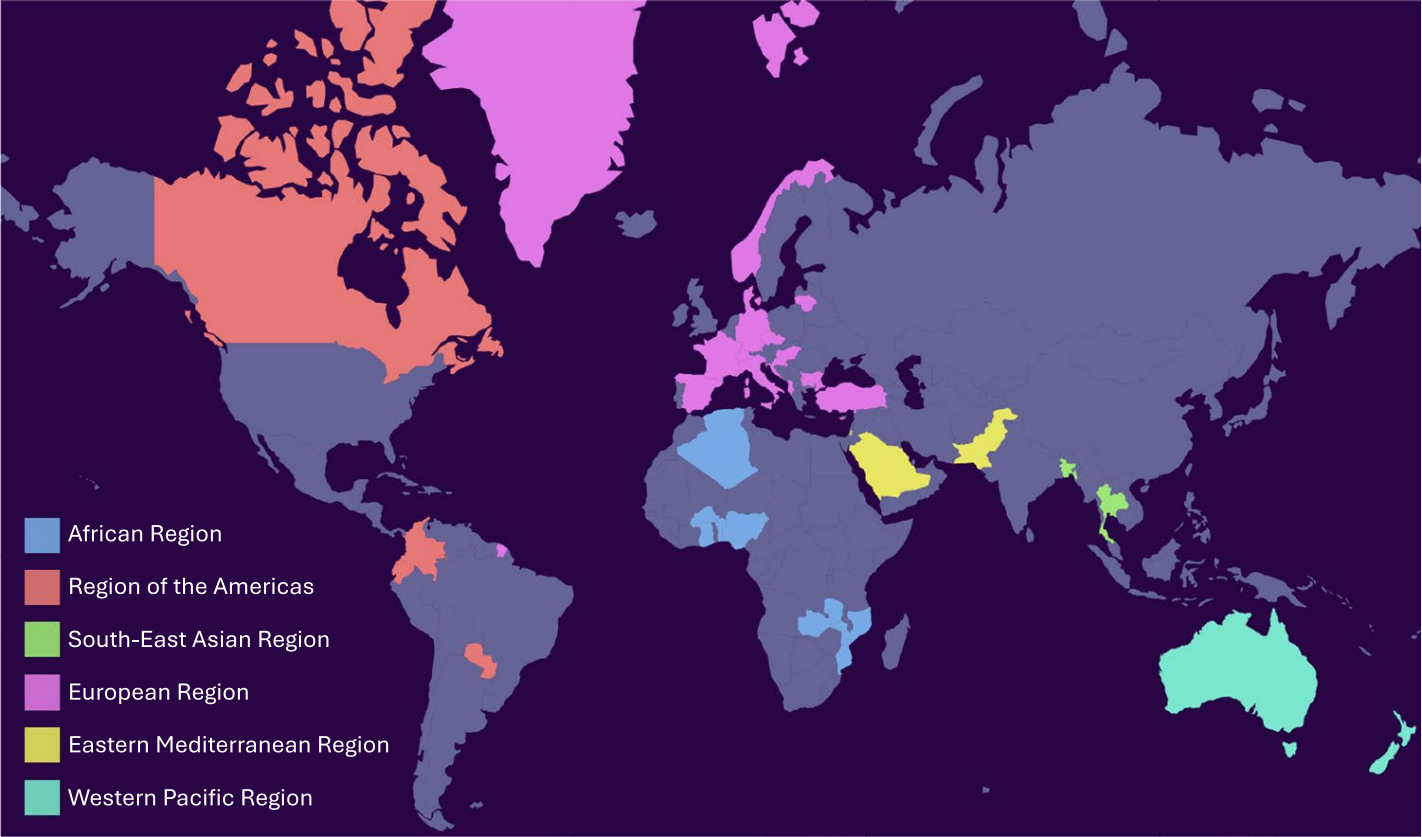
Country representation. The countries represented in the study are shown with colour coding according to the WHO-defined regions they belong to.

**Fig. 2:**
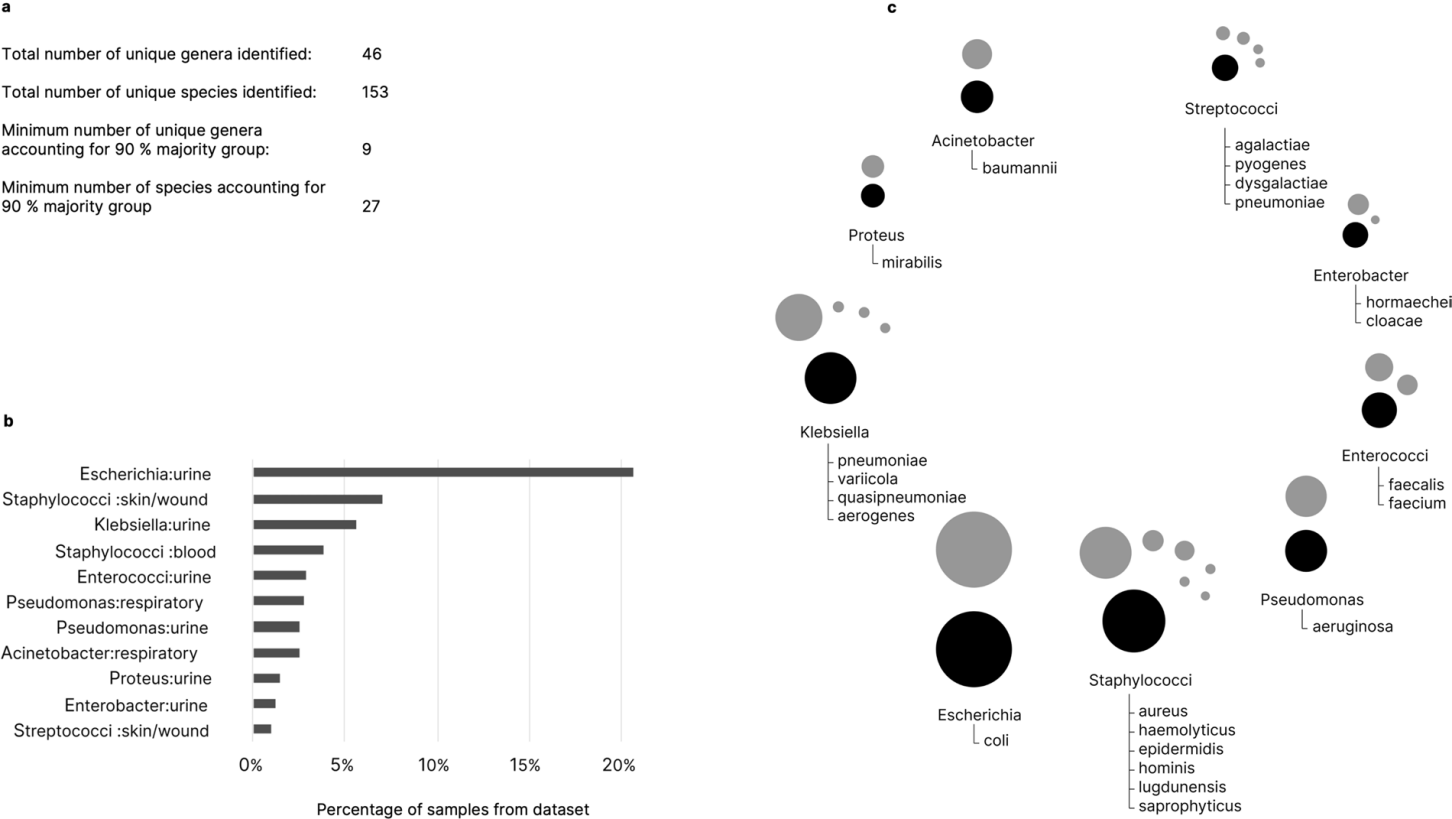
Summary description of samples in the dataset. (**a**) Number of genera identified in the dataset and accounting for the 90 percent majority groups in the dataset, (**b**) major pathogen-source combinations accounting for the majority of the dataset and (**c**) Area depiction of 9 major genera (black circles) and represented species (grey circles) in the dataset.

The presented dataset was collected and processed as part of a research project entitled “Two Weeks in the World” (TWIW), led by the Technical University of Denmark (DTU). The main purpose of the research project was to assess the species diversity and resistance gene abundance in clinically relevant pathogens across the world, in 2020. Diagnostic units involved in diagnosing causative pathogens of clinical infections (i.e. patients presenting with symptoms) from around the world, were invited to join the study. In total, 35 different countries are represented through 59 different diagnostic units. Figure 1 depicts the countries represented in the study. Summary descriptions of the dataset are depicted in Fig. 2.

**Fig. 3:**
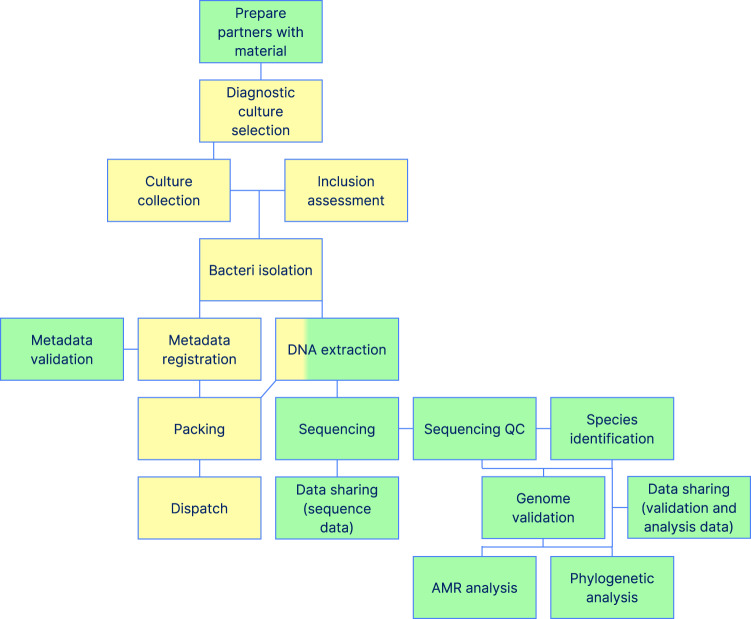
Project pipeline and framework – tasks in green were performed by DTU and tasks in yellow were performed by partners. DNA extraction was performed by some partners, who could not dispatch swabs.

Partners (i.e. diagnostic units) participated by sending either bacterial isolates or DNA extracted from bacterial isolates to Denmark (DTU). Here, isolates were cultured and DNA was extracted. All DNA (extracted by partners or by DTU) was used for whole genome sequencing (WGS) on an Illumina-based platform. Minimal metadata was required for all samples and “nice-to-know” metadata was provided by partners who were able to do so. WGS data was used to perform bioinformatic species prediction of the bacterial pathogens, identification of acquired resistance genes and inferring distance-based phylogeny. Figure 3 depicts an overview of the project pipeline and framework.

The TWIW research project can be visited through the web app: https://twiw.genomicepidemiology.org. The website allows browsing genomic insights such as phylogenetic trees.

The MySQL database is available as a “data dump“ via DTU Data 10.11583/DTU.21758456^9^ and the raw sequencing data (fastq) is available on ENA (ERP141886)^10^. The MySQL database contains information about ENA accession numbers for the sequencing data. Combined, these resources represent a complete dataset of 3087 validated bacterial genomes of clinical relevance, collected across the globe in 2020. Everything from sample origin, sequencing information, identified species, identified resistance genes, phylogenetic relationships is available and navigable through implemented relationships and table documentation in the MySQL database.

## Methods

### Preparation of partners to collect samples

Partners registered for participation by contributing isolates or DNA samples to the study. Material was sent to partners according to their registered participation format. This included material for sample collection, metadata registration, DNA extraction and sample shipment to Denmark. Specific protocols were provided, according to the registered participation format and a video for partners sampling isolates was made available via the TWIW web application and YouTube.

### Sample collection

#### Ethical considerations

Partners were in charge of navigating national guidelines and regulations regarding ethical approval (such as institutional review boards, ethical review boards or other) of their participation in the study. The Danish National Scientific Ethics Committee was consulted with regards to The Technical University of Denmark leading the study, and based on their assessment of the study protocol, the committee concluded that the samples were not human and therefore the study did not require ethical approval. No patient material was transferred with the samples, and no patient identifiers were shared with the project. Only minimal metadata pertaining to the infection and bacterial isolates or their DNA were sampled.

#### Isolate selection

Partners collected samples according to their availability to do so, during 2020. Due to the obstacles presented by the Covid-19 pandemic, ability to participate and carry out sampling was prioritised over sampling during a specific time (original study design and planning targeted sampling during March 2020).

Approximately 60 samples were collected at each individual diagnostic unit over a week. Table S1 lists the participating units with their study ID, country and city of origin, the month of collection, the amount of samples sent, whether the samples received were isolates or DNA and whether the unit made alterations to the sampling protocol. The 60 samples were to be randomly selected at the diagnostic units over the course of a week. Targeting sampling over all weekdays served the purpose of avoiding “logistical bias” from the internal logistics of the diagnostic unit. Targeting random sampling served the purpose of not targeting specific species or sample source types (i.e. urine samples, blood samples). Partners did “prospective random sampling” by estimating how many samples to collect every day over the course of a week, in order to collect approximately 60 samples over a week. Due to lack of diagnostic activities related to bacterial infections, a number of units prolonged the sampling time where simply all samples were included in the study, until 60 samples were acquired or sampling was halted due to other reasons.

#### Isolate sampling

Coal swabs were used to swab from the plates on which the pathogen was cultured — a video illustrating the isolate sampling procedure can be viewed via this link. Parafilm was strapped around the lid of the coal swab for extra sealing. Coal swabs were kept dark, at 4 °C or room temperature if 4 °C storage was not available. Swabs were stored until shipment was possible for partners.

#### DNA sampling

For partners extracting DNA, material corresponding to the DNA extraction kit and methodology used at DTU was provided to partners (DTU DNA extraction procedure is described under “DNA extraction and library preparation”). Partners were asked to provide at least 50 µl of eluted DNA, or at least 80 µl if the measured concentrations were <6 ng/µl.

#### Metadata registration

Metadata sheets were provided for all partners, together with labels with printed sample names, unique to each sampling location. Labels were for application on the samples (coal swabs or tubes with DNA) and pertaining metadata sheets. Metadata sheets were for use in a laboratory setting, where metadata could not be recorded electronically from other lab records. The collected metadata was subsequently submitted electronically via Survey Monkey or in excel format for most partners. Few partners sent only the handwritten metadata sheets. The metadata variables are listed in Table 1. Under no circumstances were internal patient identifiers (ids) or other references to individuals shared for the project.

**Table 1:** Metadata variables.

Mandatory metadata	“Nice-to-know” metadata
Geographical origin	Age of patient
Date of sampling from patient	Gender of patient
Date of sampling from lab	Hospital- or community-acquired infection
Suspected pathogen	Disease (reason for seeking health-care)
Sample source type	AMR profile as assessed by partner
	Antibiotic use history from 4 weeks prior to sampling

### Sample shipment

#### Shipping isolates

Isolates were shipped as UN3373 – biological sample category B. All coal swabs were put into absorptive pockets and into a zip lock bag labelled “UN3373”. The bag was placed in a shipment box labelled UN3373, together with any metadata sheets (these were also submitted electronically for the majority of samples). Shipment was performed by DHL, as “Medical Express” or ordinary parcel, depending on the options for the departure location. A single parcel was shipped by World Courier, from Mozambique to Denmark.

#### Shipping DNA

DNA samples were stored in Eppendorf tubes and sealed again with Parafilm. The tubes were placed in an 84-compartment foldable freezer box and placed in a bubble-wrap envelope. All DNA samples were shipped as ordinary parcels or letters, without cold chain.

### Sample handling and processing

#### Logging of received samples

Upon arrival in Denmark, samples were logged together with received metadata. Validation of the metadata was performed prior to database submission. Validation of metadata is explained in detail under “Technical Validation”. Logging entailed entering sample names (as written on the labels provided to partners), registration of unique sample id’s, original as well as validated metadata and processing information with regards to culturing and freezing of isolates. Once validated, all information resulting from logging samples and their metadata was submitted to the MySQL database.

#### Culturing of received isolates

Isolates received on coal swabs were cultured on blood agar or chocolate agar, in presence of CO2 if necessary, and sub-cultured until the expected (as submitted by sampling partner) species were (presumedly) isolated (visual recognition by experienced laboratory professionals). In doubt of which species to go forward with, multiple isolates were brought forward for DNA extraction and sequencing and the correct isolate was decided upon after bioinformatic species prediction.

#### DNA extraction and library preparation

DNA was extracted using Qiagen DNeasyÂ® Blood & Tissue kit (Qiagen, Venlo, Netherlands) according to manufacturer’s protocol. DNA concentrations were measured on Qubit using Invitrogen’s Qubit dsDNA high-sensitivity (HS) assay kit (Carlsbad, CA, USA). DNA concentrations were diluted to approximately 0.2 ng/µl for library preparation. Libraries were prepared according to the Illumina NexteraXT DNA Library Prep Reference Guide (Illumina, Inc., San Diego, CA, USA) using standard normalisation.

#### Sequencing

All samples, except eight, were sequenced on an Illumina NextSeq 500 platform, paired-end sequencing, medium output flowcell (NextSeq500/550 Mid Output Kit v2.5 300 cycles, Cat. nr 20024905). Gram-negative samples were run 96 isolates in parallel, and Gram-positive samples were run 192 isolates in parallel. Few flow cells were run with mixed Gram-negative and Gram-positive samples with approximately 100 samples on a single flow cell. Eight samples were sequenced on an Illumina MiSeq platform, paired-end sequencing, 500 cycles (2 × 251) on a V3 flowcell.

#### Data processing and analytics

Sequencing data was downloaded from BaseSpace (Illumina’s customer cloud platform) and transferred to the Danish National Supercomputer for Life Sciences^11^, a high-performance computing cluster, where it was both stored and processed, and all downstream analytics took place.

##### Raw read quality control (QC)

An in-house bioinformatics pipeline, called FoodQCPipeline v. 1.5^12^, was used at default settings to quality assess the raw sequence data, trim the raw reads according to predefined quality thresholds and perform de-novo assembly on the genomes. The quality assessment and trimming of raw sequencing data is further described under “Technical Validation”. Given the ‘–spades’ option, FoodQCPipeline performs de-novo assembly with SPAdes v. 3.11.0^13^. After running the FoodQCPipeline, both trimmed fastq data and fasta (draft assemblies) are available for downstream analyses. QC summary data was submitted to the MySQL database after genome validation, which is explained in detail under “Technical Validation”.

##### Species prediction with KmerFinder v. 3.0.2

KmerFinder^14^, was used as one of two species prediction programs. KmerFinder assesses species identity by matching k-mers from the query sequence to a kmer-based database of reference strains. KmerFinder was run on the draft assemblies with default settings, the evaluation was done on total query coverage, which is calculated as the number of unique k-mers shared between the query and the template, divided by the number of unique k-mers in the query, with the first hit being accepted if it had more than 80% total query coverage.

##### Species prediction with rMLST

The other species prediction software used, was rMLST^15^. In contrast to KmerFinder, rMLST identifies species based only on ribosomal multi-locus sequence typing, which includes the 53 genes that encode subunits of the bacterial ribosome. rMLST was run on assembled genomes through the open access API at https://pubmlst.org/species-id/species-identification-via-api. The first hit was accepted if it had more than 90% support.

##### Final species identification

The conclusion of the in silico identified species was based on either species or genus level concordance between the top hits for KmerFinder and rMLST, or an acceptable hit from only one of the two software. The point of using two different species prediction software was to allow for a sensitive assessment of whether the genomes were contaminated (KmerFinder), while complementing with a more robust but less sensitive species prediction software (rMLST). Species that could not be exactly identified are given as NA, if the genome was validated. The genome validation is described under “Technical Validation”. As with QC summary data, species prediction data was submitted to the MySQL database upon genome validation, and concordance between the KmerFinder and rmlst is given.

##### Identification of resistance-conferring genes with ResFinder 4.1

In order to identify acquired resistance genes in the validated bacterial genomes, ResFinder version 4.1^16^ was run on the assemblies. All samples were run with the ‘-s “other”’ option, meaning that the samples were not run as specific species. ResFinder has the option to run the samples as specific species, in which case a secondary program, PointFinder, is run. This analysis is omitted when running as ‘-s “other”’, and allows for complete cross-comparability of the output data resulting from our in-house ResFinder summary script, which in this case only encompasses “acquired” resistance genes. The ResFinder summary script produces different overviews of the ResFinder data, with both a class level and a drug level overview of acquired resistance genes, as well as the query coverage, percent identity to reference and position in the assembly of the hit. The ResFinder summary script is submitted as supplementary material, and is available as Supplementary file 1

##### Phylogeny

Genetic distance-based phylogeny was inferred for sequencing runs that passed the technical validation (see below), using Evergreen COMPARE^17–19^ (commit b512e6e). The reference database was the complete bacterial chromosomal genomes from the refseq collection of National Center for Biotechnology Information (NCBI), last fetched in April 2021, homology reduced to 98 percent sequence identity, using kma_index from KMA with the settings for homology reduction -hr 0.769 and -ht 0.769. Consequently, the threshold for accepting a matching reference was also lowered to 98% (76.90% k-mer identity), and the inclusion criterium for consensus sequence completeness reduced to 80%. For displaying the phylogenies on the website, a custom script (Supplementary file 2) was used to select the minimum amount of phylogenetic trees that in totality contained all possible samples.

## Data Records

The dataset consists of:

1. Raw sequence reads available at ENA: Accession ERP141886^10^
2. One MySQL database (available as MySQL data dump) for download at DTU Data, 10.11583/DTU.21758456 (URL: 10.11583/DTU.21758456.v2)^9^
3. One web application for browsing the data and selected findings, available at TWIW web app (URL: http://twiw.genomicepidemiology.org)

The Technical University of Denmark has acted as data brokers to the partners. Data brokering is the act of submitting data on behalf of another institute. This was done to ascertain that the partners would be properly referenced when the data is reused for other purposes in the future.

The MySQL database contains metadata and summary output data as well as information regarding the generation of the analysis output.

## Technical Validation

The technical validation of the dataset consists of:

1. Validation of the acquired metadata for the samples
2. Quality controlling the raw sequencing data
3. Genome validation in order for genomes to be accepted in the final dataset
4. Identification of the “correct” bacterial isolate, if several isolates were cultured from a single swab

### Validation of acquired metadata for the samples

The vast majority of partners only provided mandatory metadata (see Table 1). Metadata was submitted either via Survey Monkey, through e-mail as digital spreadsheets, or simply by sending the handwritten metadata sheets. If the information given could not be validated, no validated data was registered, in which case it is omitted from down-stream analysis. The following validations were applied to the metadata:

- Geographical origin of sample identifiable via openstreetmap.org
- Species and genus information separated, according to validated nomenclature
- Date according to specific date format (yyyy-mm-dd)
- Sample source type according to 3 validated lists: 1) type of sample, 2) anatomical origin and 3) other source indicator
- Age according to specific format (age in years)
- Gender according to specific format (‘f’, ‘m’, ‘o’)
- Hospital- or community acquired infection according to specific format (‘h’ or ‘c’)

### Quality controlling the raw sequencing data

FoodQCPipeline trims the raw reads using bbduk 2 (part of BBMap version 36.49^20^), according to three criteria: (1) the length of the read must be >50 bp, (2) phred score per base must be >20 and (3) adapters must be filtered away. FoodQCPipeline uses FastQC v. 0.11.5 to generate a quality control report for every sample.

### Genome validation

**Fig. 4:**
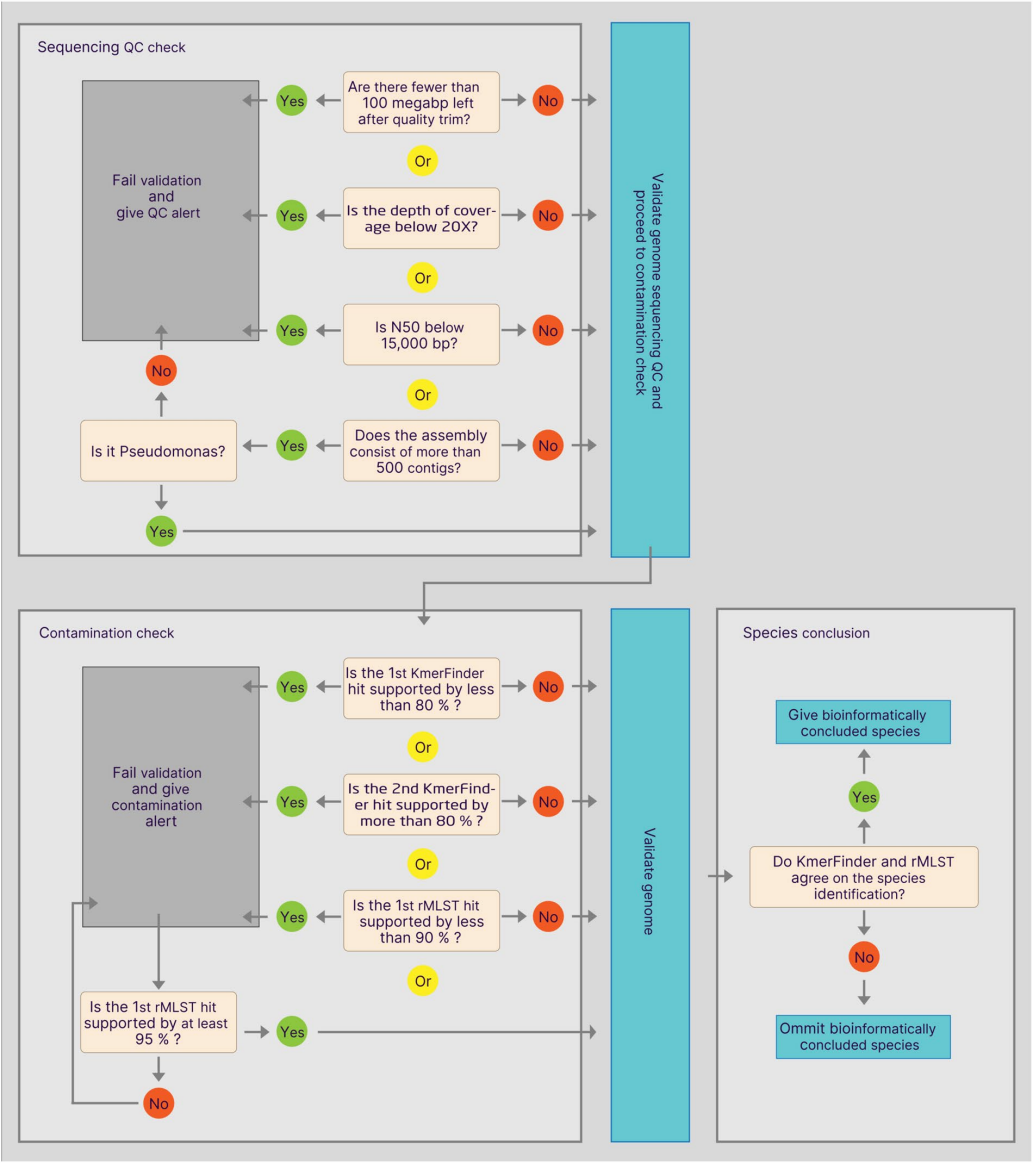
Schematic of the genome validation process employed in the qc_species_parser_v3.py.

The genome validation consists of two assessments: sequencing QC and genome contamination. The process is depicted in Fig. 4.

Based on the quality control reports generated by FoodQCPipeline, samples were discarded at the preliminary quality assessment, if the raw data did not live up to any of four criteria: (1) >100 mega bp after quality trimming, (2) depth of coverage >20X, (3) N50 > 15,000 bp and (4) <500 contigs in the assembly (unless the species prediction was a *Pseudomonas* spp., in which case up to 1098 contigs were accepted). If any of the four criteria were not met, a QC alert would be given and the genome would fail validation.

Genome contamination was assessed by the following 3 criteria:

1. the 1st KmerFinder hit had >80% total query coverage
2. that the 2nd KmerFinder hit had < than 80% total query coverage
3. that the 1st rMLST hit had >90% support.

If any of these criteria were not met, a contamination alert was given for the sample and the genome validation failed. However, in the case of a KmerFinder-based contamination alert, the genome could be validated if the rMLST 1st hit had >95% support.

All failed genomes were assessed manually afterwards, and in certain cases a genome could be manually validated based on various assessments. Reasons for manual validations (and failures) are indicated in the dataset.

### Identification of the correct bacterial isolate

When several bacterial isolates were cultured from a swab, the trained laboratory professionals attempted to correctly identify the suspected species by visual recognition. When in doubt, all isolates were brought forward for sequencing and bioinformatic analyses. In the case where one of the samples was in agreement with the suspected pathogen, this isolate was kept in the final dataset with the pertaining metadata. In the case where none of the isolates matched the suspected pathogen, they were all (typically two) kept in the final dataset with pertaining metadata and were given an “A” and “B” suffix in the sample name, but registered with unique ids.

### Exclusion reasons

Through the process from receiving samples to validating the genomes for the final dataset, reasons for samples to be excluded were:

- sample missing (some samples are registered as being received, because they were registered in the partner’s metadata, however the sample was never received/recovered)
- alternative isolate (if several isolates were cultured from a swab and another isolate matched the suspected pathogen)
- out of scope (if an isolate turned out to be something not bacteria (e.g. fungi))
- not viable
- not isolatable (typically due to insurmountable *Proteus* spp. contamination)
- contaminated with fungi (a bacterial pathogen was also present, but could not be isolated from the fungal contamination)
- x-isolate (if several isolates were cultured from a swab but the suspected pathogen was assumed visually identified and brought forward, the remaining are x-isolates)
- lab material test (if a sample was registered multiple times, simply because it was used to test laboratory material)
- not enough DNA (partners sending extracted DNA didn’t always send adequate amounts of DNA)
- contamination with no original isolate available (partners sending DNA may have sent DNA which was contaminated – in this case the original isolate could not be regrown and re-isolated)

A total 182 samples were excluded based on these reasons.

## Usage Notes

No unvalidated genomes have been submitted to ENA, and therefore it should be “safe” for data users to apply these genomes in bioinformatic analyses. The MySQL database, however, contains information regarding all received samples. Some samples could not be regrown in the laboratory in Denmark, and some DNA extracted by partners was contaminated and could not be re-extracted in Denmark, because the original isolate was not available. However, the metadata in the “sample” table in the mySQL still has information regarding what types of samples were collected from which places, as well as the suspected genus and species of the samples - even though the pertaining genomes do not exist on ENA.

### Supplementary information

Table S1

DB explainer

## Data Availability

The software used to generate the dataset is openly available either through their respective repositories linked under “Methods”, or for custom scripts, in the code repository of the project: https://bitbucket.org/genomicepidemiology/twiw_utilities/ as well as Supplementary files 1 and 2.
